# Synthesis and characterization of novel rhenium(I) complexes towards potential biological imaging applications

**DOI:** 10.1186/s13065-016-0218-4

**Published:** 2016-11-25

**Authors:** Kokila Ranasinghe, Shiroma Handunnetti, Inoka C. Perera, Theshini Perera

**Affiliations:** 1 Department of Chemistry, University of Sri Jayewardenepura, Nugegoda, Sri Lanka; 2 Institute of Biochemistry, Molecular Biology and Biotechnology, University of Colombo, Colombo, Sri Lanka; 3 Department of Zoology and Environmental Sciences, University of Colombo, Colombo, Sri Lanka

**Keywords:** Rhenium tricarbonyl, NMR spectroscopy, Cytotoxicity, Fluorescent

## Abstract

**Background:**

Re(I) tricarbonyl complexes exhibit immense potential as fluorescence imaging agents. However, only a handful of rhenium complexes have been utilized in biological imaging. The present study describes the synthesis of four novel rhenium complexes, their characterization and preliminary biological studies to assess their potential as biological imaging agents.

**Results:**

Four facial rhenium tricarbonyl complexes containing a pyridyl triazine core, (L1 = 5,5′(3-(2-pyridyl)-1,2,4-triazine-5,6-diyl)-bis-2-furansulfonic acid disodium salt and L2 = (3-(2- pyridyl)-5,6-diphenyl-1,2,4-triazine-4′,4′′-disulfonic acid sodium salt) have been synthesized by utililzing two different Re metal precursors, Re(CO)_5_Br and [Re(CO)_3_(H_2_O)_3_]OTf in an organic solvent mixture and water, respectively. The rhenium complexes [Re(CO)_3_(H_2_O)L1]^+^ (**1**), Re(CO)_3_L1Br (**2**), [Re(CO)_3_(H_2_O)L2]^+^ (**3**), and Re(CO)_3_L2Br (**4**), were obtained in 70–85% yield and characterized by ^1^H NMR, IR, UV, and luminescence spectroscopy. In both H_2_O and acetonitrile, complexes display a weak absorption band in the visible region which can be assigned to a metal to ligand charge transfer excitation and fluorescent emission lying in the 650–710 nm range. Cytotoxicity assays of complexes** 1**,** 3**, and** 4** were carried out for rat peritoneal cells. Both plant cells (*Allium cepa* bulb cells) and rat peritoneal cells were stained using the maximum non-toxic concentration levels of the compounds, 20.00 mg ml^−1^ for** 1** and** 3** and 5.00 mg ml^−1^ for** 4** to observe under the epifluorescence microscope. In both cell lines, compound concentrated specifically in the nuclei region. Hence, nuclei showed red fluorescence upon excitation at 550 nm.

**Conclusions:**

Four novel rhenium complexes have been synthesized and characterized. Remarkable enhancement of fluorescence upon binding with cells and visible range excitability demonstrates the possibility of using the new complexes in biological applications.

**Graphical abstract:**
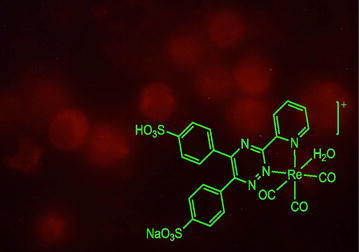
Micrograph of rat peritoneal cells incubated with novel rhenium complex under epifluorescence microscope.

**Electronic supplementary material:**

The online version of this article (doi:10.1186/s13065-016-0218-4) contains supplementary material, which is available to authorized users.

## Background

Metal complexes possess unique properties such as radioactivity [1, 2], preferential binding to certain proteins or organelles [3–7], inertness [8], lower toxicity than the purely organic molecules [9] and special photophysical properties [10–13] which make them eligible for both therapeutic and diagnostic applications [14–18]. Two-photon absorption behavior of certain transition metal complexes containing conjugated ligands show high applicability in biological imaging [19, 20]. Specifically, rhenium(I) metal complexes have attracted special attraction over other metals as their chemical characteristics demonstrate better potentiality for biochemical applications [20–22]. Longer life times [13, 14], high photostability [7, 20] and large Stoke’s shifts [7, 23] make them ideal candidates for either in vitro or in vivo visualization of biological processes [24, 25].Their visible light excitation minimizes the UV damage to cells whereas conjugation with proteins and lipids facilitate their compatibility with biological systems [26]. Since Re(I) has d^6^ electronic configuration at the outer most shell, it possesses a low spin coordination sphere in metal–ligand complexes. This spatial structure of the metal coordination sphere makes the Re metal ion kinetically inert towards ligand substitutions which mitigate the metal-DNA interactions [20, 26], hence heavy metal toxicity. In addition to the kinetic inertness, the common availability of the robust *fac*-[Re(CO)_3_]^+^ core as air stable *fac*-[Re(CO)_3_(H_2_O)_3_]^+^ has been identified as an advantage for target-specific radiopharmaceutical synthesis, since aqua ligands can be easily substituted by a variety of functional groups such a amines, phospines and thioles [1, 27].

Fluorescence imaging is a nondestructive method [28], and noted over other in vitro visualization methods due to not only the increasing availability of various biocompatible fluorophores [29] but also due to its features such as sensitivity [28] and spatial resolution [10]. The ability to visualize in vitro biological processes not only in individual live cells but also in sub cellular components [30] such as DNA [28], exemplify fluorescence staining among other imaging techniques. Many Re(I) carbonyl complexes synthesized in recent years exhibit luminescent properties [7, 14, 20–24, 26] which is believed to originate from the metal-to-ligand charge transfer (MLCT) transitions [20–22, 28]. As an example, many rhenium(I) polypyridine complexes studied by Lo et al. exhibit triplet metal-to-ligand charge transfer emission [7, 21, 31, 32]. Since these transitions are partially forbidden, the decay times for fluorescence occurring from Re(I) complexes are longer [28], which then makes them easily distinguishable from autofluorescence of the biological substances, the obstacle for many well-known fluorescent probes [26]. Furthermore, the larger Stoke’s shifts and higher photostability of these metal complexes create the opportunity to prevent probe–probe overlapping which enables staining different subcellular components simultaneously [28]. In addition, experiments on molecular dynamics in microsecond timescale are now possible due to polarized emission ability [23, 28] of transition metal complexes such as Re.

The coordination chemistry of both Re and ^99m^Tc are similar and therefore Re metal–ligand complexes serve as model systems for ^99m^Tc-ligand complexes [1, 27, 33, 34] which enables correlation between in vitro and in vivo imaging. This correlation has led to a pathway to understand the behavior of radiopharmaceuticals at subcellular levels [23]. Several other correlations [35, 36] originating from Re(I) metal complexes containing pharmaceuticals as ligands, are under investigation and successful concepts such as “single core multimodal probes” [11] have been established. Furthermore, beta emitting Re isotopes such as Re^188^ and Re^186^ possess the possibility to serve in therapeutic applications [33], thereby increasing the importance of structural and spectral characterization of novel complexes of the non radioactive istotope of rhenium.

**Fig. 1:**
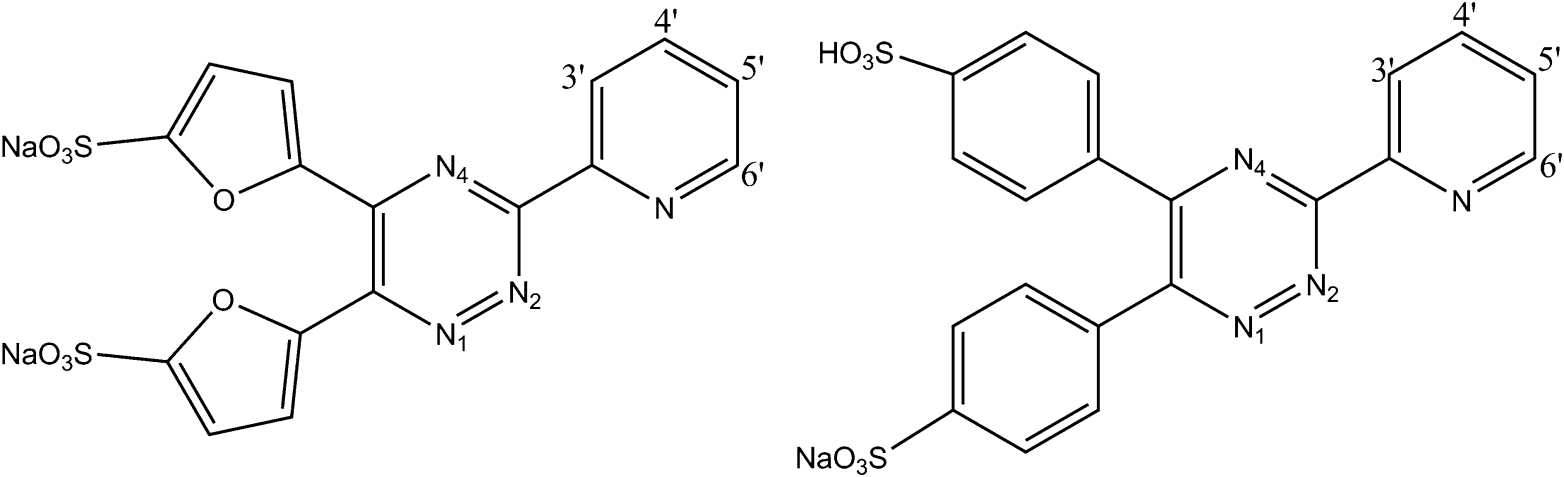
Chemical structures of 5,5′(3-(2-pyridyl)-1,2,4-triazine-5,6-diyl)-bis-2-furansulfonic acid disodium salt (L1, *left*), and 3-(2-Pyridyl)-5,6-diphenyl-1,2,4-triazine-4′,4′′-disulfonic acid sodium salt (L2, *right*)

**Fig. 2:**
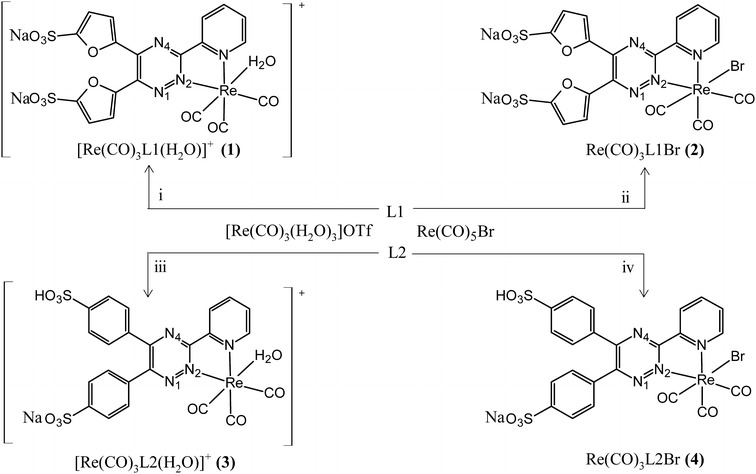
Synthetic routes of complexes. (*i*) 4 h reflux in 10:1 acetonitrile:water mixture (*ii*) 0.16 h reflux in water (*iii*) 0.16 h reflux in water (*iv*). 8 h reflux in 7:2:1 acetonitrile:methanol:water mixture

During this study two water soluble ligands having conjugated aromatic systems, 5,5′(3-(2-pyridyl)-1,2,4-triazine-5,6-diyl)-bis-2- furansulfonic acid disodium salt (L1) and 3-(2-pyridyl)-5,6-diphenyl-1,2,4-triazine-4′,4′′- disulfonic acid sodium salt (L2) were utilized (Fig. 1), with the objective of promoting the permeability of complexes into cellular membranes. The hydrophilicity was retained to some extent by choosing their anionic form which made synthesis feasible in polar solvents. We report here the synthesis of four novel complexes utilizing two different rhenium precursors as illustrated in Fig. 2.

Even though various metal complexes have been synthesized, characterized, and identified in recent years, their potential applicability as fluorophores and their practical use as biochemical probes are at an infant stage due to limitations such as bio toxicity. Therefore, cytotoxicity of the synthesized compounds was analyzed for mammalian cells and the ability to act as microscopy stains were tested in both plant and mammalian cells.

## Results and discussion

### Synthesis and spectroscopic properties

Four rhenium tricarbonyl complexes containing L1 and L2 were synthesized (Fig. 2) in good yield by utilizing two different Re metal precursors, Re(CO)_5_Br and [Re(CO)_3_(H_2_O)_3_]OTf, in an organic solvent mixture and in water, respectively.

The spectroscopic data obtained for each complex confirm the extent of purity of complexes as well as their photophysical properties. Strong peaks in the 2035 to 1880 cm^−1^ range in FTIR spectra obtained for metal complexes are characteristic to the three carbonyl peaks in the metal coordination sphere and confirm the presence of the *fac*-Re(CO)_3_^+^ core [1]. A broad peak is obtained for complex Re(CO)_3_L1(H_2_O)]^+^ (**1**) at 1889 cm^−1^ indicating overlap of peaks as previously reported for similar Re(I)(CO)_3_L complexes where L = ethyl (bis(2-pyridylmethyl)amino)acetate [1] and L = 2,4,6-tris(2-pyridyl)-1,3,5-triazine [37]. The Re(CO)_5_Br metal precursor contains three peaks for vibrational stretching of carbonyl ligands in the 2034 to 1976 cm^−1^ range and the formation of complex **2** has shifted the collection of peaks to lower energy levels due to changes in the chemical environment. Similar shifts were observed in IR spectra of all four metal complexes compared to their metal precursors, which confirm the formation of novel bonds with ligands.

**Fig. 3:**
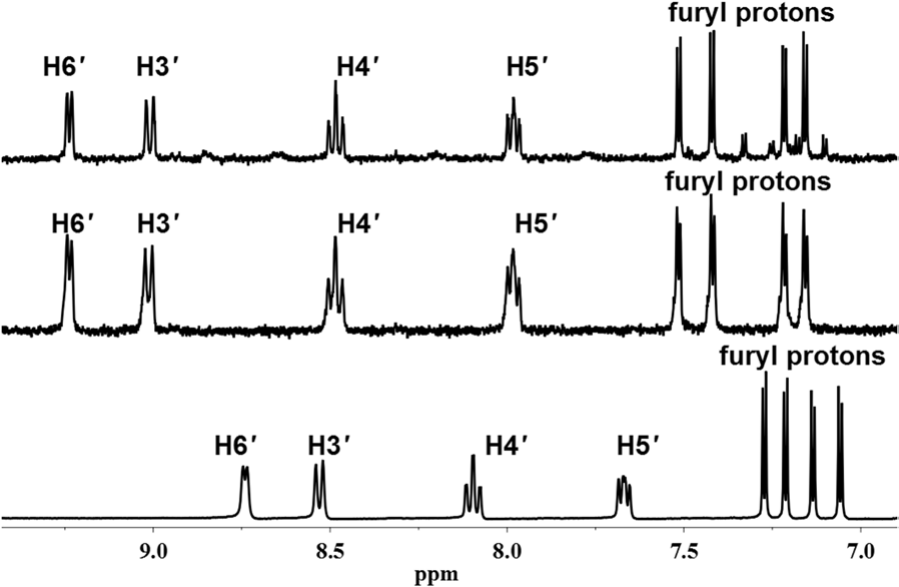
^1^H NMR spectra of L1 (*bottom*), [Re(CO)_3_L1(H_2_O)]^+^ (**1**) (*middle*) and Re(CO)_3_L1Br (**2**) (*top*) in D_2_O at 25 °C

Further, purity of the dried residues of the complexes and ligands were confirmed by ^1^H and ^13^C NMR data. The assignment of signals was based on the chemical shifts, coupling patterns and splitting patterns of each peak. These assignments were further confirmed by the data from 2D NMR experiments for complexes **1** and **3** (Additional file 1). The significant difference between the spectra of the uncoordinated ligands and their rhenium bound complexes is the deshielding of the peaks, which is expected to be higher for protons closer to the metal atom, due to electron withdrawing inductive effects of Re(I). In the free ligand (L1/ferene), the pyridyl H6′ signal is the most downfield doublet (8.85 ppm) consistent with its close proximity to pyridyl nitrogen. In the spectrum of the metal complex **1**, the H6′ signal appears even more downfield (9.24 ppm, Fig. 3) which confirms the metal-N1′ bond formation. Several previously reported examples have illustrated the ability of Re(I) metal ion to form five membered rings with ligands containing the bipyridyl core [12, 26, 38]. The same ring formation, without any rotational confirmations has been observed between Pt and ligands containing the pyridyl triazine core, of which the chemical structures have been confirmed by crystallographic data [8]. Thus, the metal complexes of this study were expected to bond with ligands by forming five membered rings with N2 and N1′ nitrogen atoms. All the proton peaks of the ligand were further deshielded upon bond formation with Re(I) ion in complex **1** (Fig. 3) and support the proposed chemical structure. Fural protons give four closely spaced doublets within the 7.10–7.34 ppm range which also shift down field (7.15–7.52 ppm) upon metal bonding; however assignment of them by only using this information is not prudent and thus the fural signals have been collectively assigned for the purpose of this study. The spectra for complexes [Re(CO)_3_L1(H_2_O)]^+^ (**1**) and Re(CO)_3_L1Br (**2**) bearing the same ligand, are similar except (almost negligible) extra peaks due to the presence of trace amounts of solvents and excess ligand in complex **2**. The coordination of bromide in complex **2** has been confirmed by ESI mass spectrometric analysis.

**Fig. 4:**
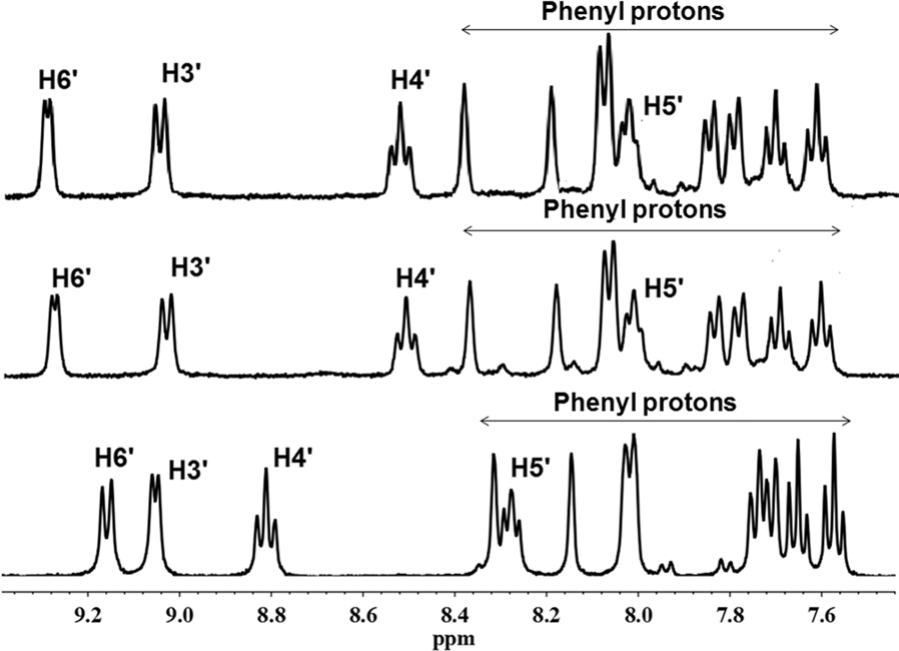
^1^H NMR spectra of L2 (*bottom*), [Re(CO)_3_L2(H_2_O)]^+^ (**3**) (*middle*) and, Re(CO)_3_L2Br (**4**) (*top*) in D_2_O at 25 °C

Even though the ^1^H NMR spectrum of L2 (Fig. 4) is comparatively more complicated due to the presence of phenyl rings, the expected chemical shifts over close proximity to pyridyl nitrogen were seen in a spectrum of the free ligand. The ^1^H NMR spectra for complexes [Re(CO)_3_L2(H_2_O)]^+^ (**3**) and Re(CO)_3_L2Br (**4**) are very much similar to each other and the highest deshielding is exhibited by H6′ and H3′ as expected. This further deshielding can be attributed to the reduction of electron density in vicinity due to the bond formation of Re with pyridyl N atom. Unusual upfield shifts of the proton peaks attributed to H4′ (8.53 and 8.51 ppm) and H5′ (8.04 and 8.01 ppm) (Fig. 4) were observed in complexes **3** and **4**, respectively, in comparison with that of the uncoordinated ligand (H4′: 8.81 ppm and H5′: 8.27 ppm). Shielding of H5′ and H4′ protons upon metal–ligand bond formation may have occurred due to ring current effects or steric effects of the phenyl rings which tilt the N-Re–N plane upon N coordination to Re. These upfield shifts were not observed in complexes with L1 which had fural rings (Fig. 3). However, upon coordination to Re, the H4′ and H5′ protons appear around similar values in all four complexes, irrespective of having fural or phenyl groups (Additional file 1: Table S1).

### UV visible and luminescence spectroscopy

UV visible absorption spectra of all four complexes and of the two free ligands were measured in water at room temperature. Absorption spectra for uncoordinated ligands showed isolated bands at 342–325 nm for L1 and L2, respectively due to ligand centered transitions. The metal complexes showed two broad absorptions at comparatively longer wavelengths (Table 1, Additional file 1: Figures S1 and S2 of UV–VIS spectra in Additional file) in comparison with free ligands. The four new rhenium complexes fall into the special category, Metal–Ligand complex (MLCs) [28, 39]. According to previously reported studies, MLCs usually show closely associated, MLCT bands which are lower in energy than inter-ligand transitions (IL) [13, 39–43]. The absorption spectra of the new complexes are in agreement with this observation (Table 1); therefore the low energy bands for each complex can be assigned as MLCT. The emission spectra obtained for the new complexes show weak fluorescent bands in the visible region. The visible range excitability of the novel complexes promises lesser damage in biological applications, when compared to most of the modern fluorescent imaging agents which need to be excited in the UV range.

**Table 1:** Electronic, emission spectral data of complexes **1**–**4** in H_2_O at 25 °C

Complex	UV visible absorption/nm		Excitation/nm	Emission/nm
	Inter-ligand	MLCT		
^w^[Re(CO)_3_L1(H_2_O)]^+^ (**1**)	330	420	425	700
^a^[Re(CO)_3_L1(H_2_O)]^+^ (**1**)			470	710
^w^Re(CO)_3_L1Br (**2**)	328	400	400	480^b^, 658
^w^[Re(CO)_3_L2(H_2_O)]^+^ (**3**)	315	395	441	672
^a^[Re(CO)_3_L2(H_2_O)]^+^ (**3**)			424	645
^w^Re(CO)_3_L2Br (**4**)	300	396	398	640
^a^Re(CO)_3_L2Br (**4**)			396	645

^W^In water

^a^In acetonitrile

^b^Peak due to excess ligand

### Bio-molecular probing ability

Complexes, [Re(CO)_3_L1(H_2_O)]^+^ (**1**), [Re(CO)_3_L2(H_2_O)]^+^ (**3**), and Re(CO)_3_L2Br (**4**) are highly soluble in both water and PBS-BSA medium which makes them eligible to be used in in vitro biological experiments. Each complex was tested for cytotoxicity using Trypan blue staining method and none of them were considerably toxic to rat peritoneal cells up to reasonable concentrations which is a desired character of a biological imaging agent. Complexes **1** and **3** are nontoxic up to 20.00 mg/ml concentrations. However Re(CO)_3_L2Br (**4**) showed relatively higher toxicity than complexes **1** and **3**. This excessive toxicity may be attributed to the presence of Br atom in complex Re(CO)_3_L2Br (**4**), instead of a H_2_O molecule as in [Re(CO)_3_L1(H_2_O)]^+^ (**1**) and [Re(CO)_3_L2(H_2_O)]^+^ (**3**). Compounds bearing halogen groups have been reported to demonstrate higher toxicity when compared to the non-halogenated analogues [44]. We attribute the increased cytotoxicity of complex **4** to its increased lipophilicity in comparison with that of complex **3**.

In order to confirm the potential use of these Re complexes as fluorophores, their ability to act as microscopic stains was tested using plant cells (A*llium cepa* bulb cells) and rat peritoneal cells. Complexes were seen to be selectively bound to the nuclear region in the cells. Even though the complexes have shown weaker fluorescence in water itself, it has given sharp fluorescence images under the epifluorescence microscope system. We attribute this to increased conjugation or structural rigidity [45] after binding with cells which may have enhanced the fluorescence yield. According to Olmstead and co-workers [46], the fluorescent enhancement of certain substances upon binding occurs due to reduction of the rate of excited proton transfer to solvent molecules. However, further work should be carried out to confirm the exact reason of observed fluorescent enhancement.

### In vitro cytotoxicity

**Fig. 5:**
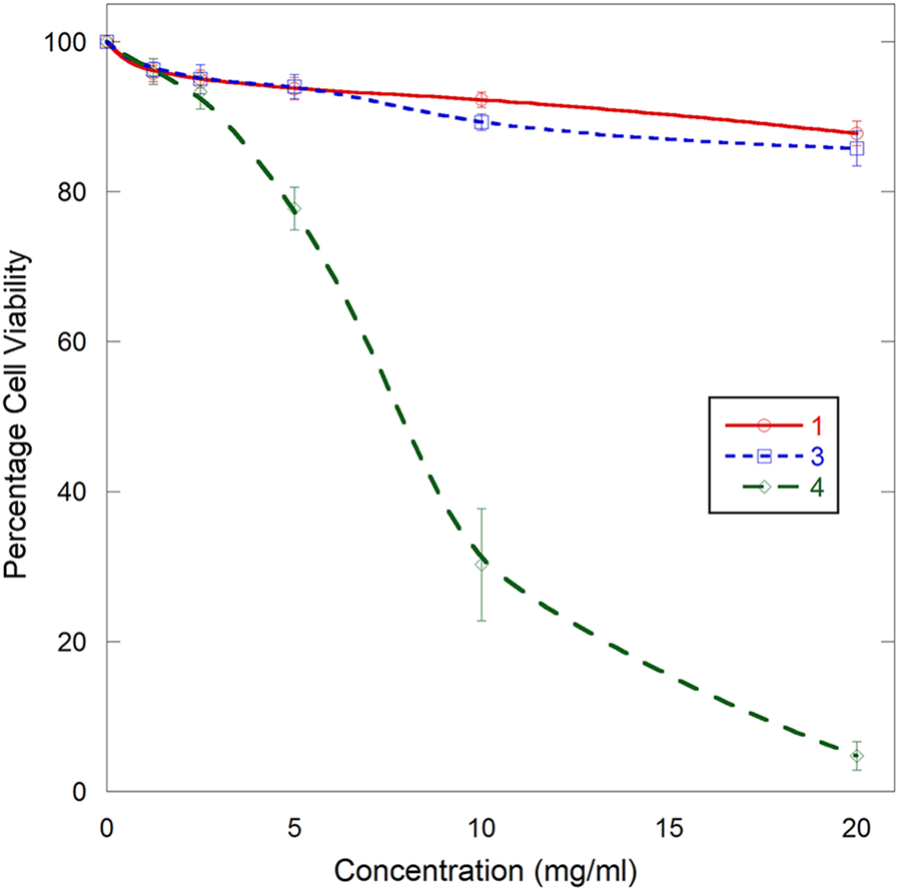
Percentile viability of rat peritoneal cells incubated in compounds [Re(CO)_3_L1(H_2_O)]^+^(**1**), [Re(CO)_3_L2(H_2_O)]^+^ (**3**) and Re(CO)_3_L2Br (**4**) at different concentrations

There was no significant toxicity observed up to 20.00 mg/ml concentrations of complexes [Re(CO)_3_L1(H_2_O)]^+^ (**1**) and [Re(CO)_3_L2(H_2_O)]^+^ (**3**) in which the cell viability was in the range of 96 to 85% throughout the considered concentration range. However, complex Re(CO)_3_L2Br (**4**) was not tolerated by rat peritoneal cells at higher concentrations than 5.00 mg ml^−1^ at which the viability is 77% (Fig. 5).

**Fig. 6:**
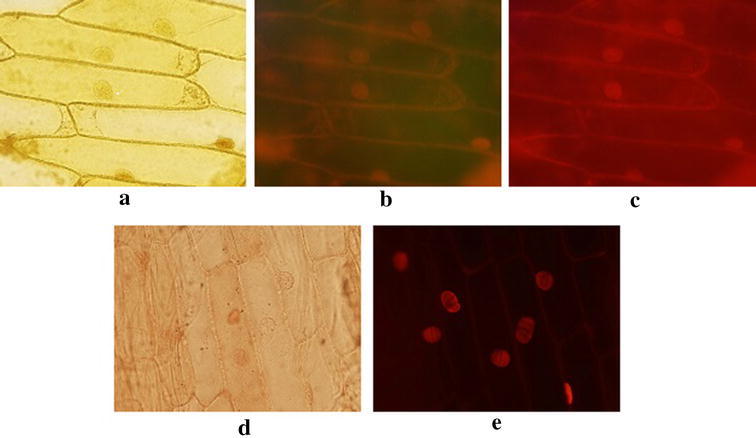
*Allium Cepa* bulb cells incubated with 20.00 mg ml^−1^ of [Re(CO)_3_L2(H_2_O)]^+^ (**3**) in PBS-BSA solution under optical micrograph (**a**). Fluorescence micrographs of same cells excited at 450 nm (**b**), excited at 550 nm (**c**). *Allium Cepa* bulb cells incubated with ethidium bromide in PBS-BSA solution under optical micrograph (**d**). Fluorescence micrographs of same cells excited at 450 nm (**e**)

Illumination of plant cells incubated with [Re(CO)_3_L2(H_2_O)]^+^ (**3**) at 450 nm (blue color) resulted in weaker fluorescence images when compared to images taken at 550 nm (Fig. 6). This deviation from the results obtained by photo physical properties (MLCT excitation at 424 nm) indicate that a novel binding mode may be involved between the complex and the cellular environment which has altered its fluorescent nature. Since the ligand itself does not result in any fluorescence image upon illumination at any of the above two wavelengths, it may be concluded that the novel binding of the metal complex with cells and also the enhanced luminescent properties originate originating from that binding occur solely due to the transition metal complex and not due to the ligand. Thus, [Re(CO)_3_L1(H_2_O)]^+^ (**1**), [Re(CO)_3_L2(H_2_O)]^+^ (**3**) and Re(CO)_3_L2Br (**4**) are suitable not only as biological imaging agents but also as model systems for ^99m^Tc complexes to enable complementary fluorescent and radioactive probe pairs which correlate in vitro and in vivo imaging studies.

**Fig. 7:**
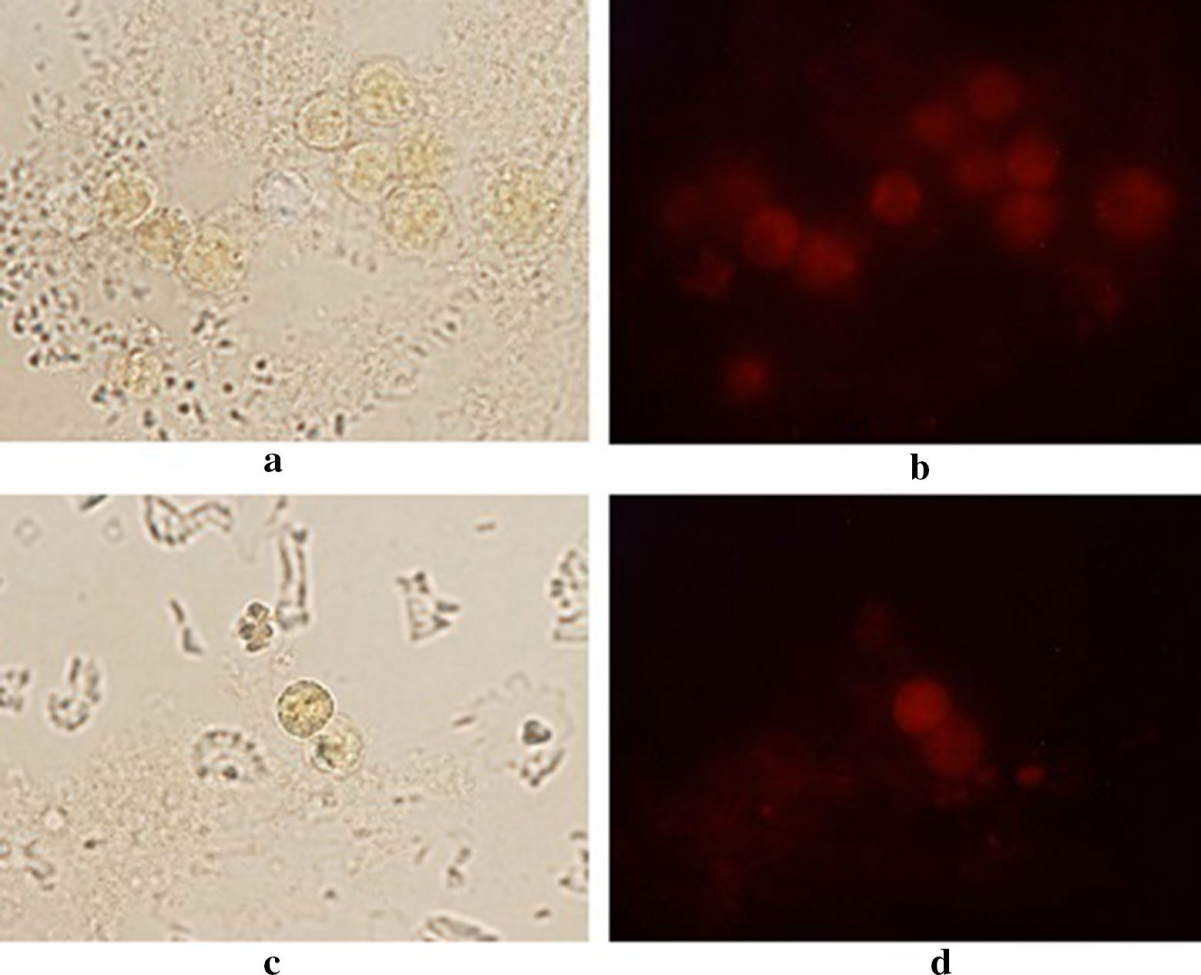
Micrographs of rat peritoneal cells incubated with 20.00 mg ml^−1^ of [Re(CO)_3_L2(H_2_O)]^+^ (**3**) in PBS-BSA solution under optical microscope (**a**), under epifluorescence microscope (**b**). Micrographs of rat peritoneal cells incubated with 20.00 mg ml^−1^ of [Re(CO)_3_L1(H_2_O)]^+^ (**1**) in PBS-BSA solution under optical microscope (**c**),under epifluorescence microscope (**d**)

The metal complexes are seen to associate with nuclei and this observation is confirmed by the images of stained plant cells in which only the nuclei show fluorescence (Fig. 6). Since rat peritoneal cells possess relatively larger nuclei the micrographs show gleaming of whole cells (Fig. 7).

Even though the compound [Re(CO)_3_L1(H_2_O)]^+^ (**1**) has not shown relatively good photo physical properties in solution, after binding with cells its conjugation may have altered to result in better fluorescence properties. Ethidium bromide, a well-known fluorophore, was used as the positive control within the experiment. Even though these complexes do not give as sharp images as the positive control (Fig. 6), adequate amount of imaging potential can be seen in all three compounds.

## Experimental section

### Starting materials

5,5′(3-(2-pyridyl)-1,2,4-triazine-5,6-diyl)-bis-2-furansulfonic acid disodium salt (ferene/L1), 3-(2-pyridyl)-5,6-diphenyl-1,2,4-triazine-4′,4′′-disulfonic acid sodium salt (L2), Re(CO)_10_, bromine water and AgOTf were obtained commercially from Sigma Aldrich and Re(CO)_5_Br and [Re(CO)_3_(H_2_O)_3_]OTf (OTf = trifluoromethanesulfonate) were prepared by known methods [47]. A 0.1 M solution of [Re(CO)_3_(H_2_O)_3_]OTf was used for the synthesis of the metal complexes and was prepared by carefully weighing 0.238 g of [Re(CO)_5_OTf] directly into the reaction vial, into which exactly 5000 μl of water was pipetted out and heated at reflux for 30 min. Analytical grade water and methanol purchased from Merck Specialties (Pvt) Limited and used as received. Carrageenan (Commercial grade-Type I) and Bovine Serum Albumin (BSA) were purchased from Aldrich and used as received. Phosphate buffered saline (1X PBS), 1 mg/ml PBS-BSA solution and 0.2% Trypan blue were prepared by known methods [48]. Healthy, white albino rats were selected from the animal house of the Department of Zoology and Environment Sciences, University of Colombo, Sri Lanka. Ethical clearance for extracting animal cells was obtained from the Research, Ethics and Higher Degrees Committee of the Institute of Biochemistry, Molecular Biology and Biotechnology of the University of Colombo and the experiments were performed according to internationally accepted guidelines for handling laboratory animals.

### NMR measurements

^1^H, ^13^C, ^1^H-^1^H ROESY, and ^1^H-^13^C HSQC (400 MHz) NMR spectra were recorded in D_2_O on a Bruker spectrometer and all peak positions are relative to TSP. NMR data were processed with Mestre-C software.

### Mass spectrometric measurements

High resolution mass spectra were recorded on an Agilent 6210 ESI TOF LCMS mass spectrometer.

### Synthesis of complexes

#### [Re(CO)_3_L1(H_2_O)]OTf (**1**)

A solution of [Re(CO)_3_(H_2_O)_3_]OTf (1 ml, 0.1 mmol) and L1 (0.0494 g, 0.1 mmol) in water (5 ml) was refluxed for 16 h. The resulting clear and bright red solution was cooled to room temperature and its volume reduced to give a fine red precipitate which was collected on a filter and dried (0.063 g 83% yield).^1^H NMR (ppm) in D_2_O: 9.24 (d, H6′), 9.02 (d, H3′), 8.49 (t, H4′), 7.98 (t, H5′), 7.52-7.15 (fural H). ^13^C NMR (ppm) in D_2_O: 199.2–193.6 (CO), 166.6–148.3 (triazine C), 157.7 (C6′), 130.1 (C3′), 144.3 (C4′), 133.4 (C5′), 116.5–121.2 (fural C). IR (cm^−1^): 2031, 1889 (CO). UV Vis (nm, in H_2_O): 330, 420. ESI–MS (*m/z*): [M]^−^ calcd for C_19_H_10_N_4_O_12_ReS_2_, 734.9272; found, 734.9271.

#### Re(CO)_3_L1Br (**2**)

A solution of Re(CO)_5_ Br (0.0406 g, 0.1 mmol) and L1 (0.0494 g, 0.1 mmol) in acetonitrile (50 ml) and water (5 ml) was heated at reflux for 4 h. The resulting clear and deep red solution was cooled to room temperature and upon reducing its volume yielded a fine deep red precipitate which was collected on a filter and dried (0.060 g, 72% yield). ^1^H NMR (ppm) in D_2_O: 9.25 (d, H6′), 9.02 (d, H3′), 8.48 (t, H4′), 7.99 (t, H5′), 7.52–7.16 (fural H). ^13^C NMR (ppm) in D_2_O: 200.1–193.6 (CO), 166.2–148.1 (triazine C), 157.6 (C6′), 130.2 (C3′), 144.3 (C4′), 133.3 (C5′), 116.5–126.0 (fural C). IR (cm^−1^): 2022, 1920, 1887 (CO). UV Vis (nm, in H_2_O) 328, 400. ESI–MS (*m/z*): [M]^−^ calcd for C_19_H_9_BrN_4_O_11_ReS_2_, 796.8428; found, 796.8394.

#### [Re(CO)_3_L2(H_2_O)]OTf (**3**)

A solution of [Re(CO)_3_(H_2_O)_3_]OTf (1.000 ml, 0.1 mmol) and L2 (0.0508 g, 0.1 mmol) in water (5 ml) was refluxed for 16 h. The resulting clear solution was cooled to room temperature and its volume reduced to give reddish orange crystals (0.053 g, 70%). ^1^H NMR (ppm) in D_2_O: 9.29 (d, H6′), 9.05 (d, H3′), 8.53 (t, H4′), 8.05 (t, H5′), 8.39–7.62 (phenyl H).). ^13^C NMR (ppm) in D_2_O: 199.2–193.6 (CO), 167.4–146.6 (triazine C), 157.5 (C6′), 130.5 (C3′), 144.5 (C4′), 132.5 (C5′), 129.7–136.9 (phenyl C). IR (cm^−1^): 2023, 1897 (CO).UV Vis (nm, in H_2_O) 315, 395. ESI–MS (*m/z*): [M]^−^ calcd for C_23_H_14_N_4_O_10_ReS_2_, 754.9686; found, 754.9695.

#### Re(CO)_3_L2Br (**4**)

A solution of Re(CO)_5_ Br (0.0406 g, 0.1 mmol) and L2 (0.0508 g, 0.1 mmol) in a mixture of acetonitrile (35 ml), methanol (10 ml) and water (5 ml) was heated at reflux for 8 h. The resulting clear solution was cooled to room temperature and deep red crystals were obtained upon reducing its volume (0.069 g, 82% yield). ^1^H NMR (ppm) in D_2_O: 9.27 (d, H6′), 9.03 (d, H3′), 8.51 (t, H4′), 8.01 (t, H5′), 8.08–7.58 (phenyl H). ^13^C NMR (ppm) in D_2_O: 199.8–193.5 (CO), 166.2–148.1 (triazine C), 157.5 (C6′), 130.4 (C3′), 144.5 (C4′), 132.6 (C5′), 129.7–136.9 (phenyl C). IR (cm^−1^): 2019, 1888 (CO). UV Vis (nm, in H_2_O) 300, 396. ESI–MS (*m/z*): [M]^−^ calcd for C_23_H_13_BrN_4_O_9_ReS_2_, 816.8842; found, 816.882.

### Photoluminescence measurements

Emission spectra were recorded on a Thermoscientific Lumina Fluorescence spectrometer, using a 150 W Xenon Lamp as the excitation source. Data were processed with Luminous software.

### In vitro cytotoxicity assays

Isolation of rat peritoneal cells was done as described previously [49]. Viability of the mammalian cells upon incubation in a mixture of 1 mg ml^−1^ PBS-BSA with each aqueous solution of complexes (due to the presence of trace amounts of solvent and excess ligand, complex **2** was not used in biological studies) for 30 min at 37 °C was determined by the Trypan blue dye exclusion method using a hemocytometer (Neubauer-Germany). Viability of rat peritoneal cells in solutions of metal complexes at different concentrations were calculated with respect to the cell viability of the control sample and represented as the percentage of living cells ± SEM (Standard Error of the Mean) where sample size is 4 (each experiment was repeated and each sample counting was done in duplicates).

### Fluorescence micrographs

Stained plant and mammalian cells by incubating them in maximum tolerable concentrations (20 mg ml^−1^ solutions of complexes [Re(CO)_3_L1(H_2_O)]^+^ (**1**) and [Re(CO)_3_L2(H_2_O)]^+^ (**3**), 5 mg ml^−1^ solution of Re(CO)_3_L2Br (**4**)) of aqueous solutions of complexes for 10 min at room temperature were observed under both optical and Olympus BX51 epifluorescence microscopes. Fluorescent micrographs were obtained with the aid of Olympus DP70 and analyzed using Olympus Stream software.

## Conclusions

Four rhenium complexes which showed good chemical stability in solution have been synthesized in good yield. NMR spectral characterization was utilized to ascertain the purity of complexes. Further characterization was done using UV–VIS, FTIR and emission spectra of all four complexes. The metal–ligand bond formation was clearly corroborated using UV–VIS absorption spectra since all four complexes exhibit an additional absorption band compared to ligand spectra which was assigned for MLCT transitions. These MLCT absorptions lie in 390 to 420 nm range. In addition FTIR spectra also provided supportive evidence for their purity and chemical stability with time. Photo physical properties indicate the fluorescent ability of complexes. Each complex showed emission within visible range from 600 to 700 nm providing large Stoke’s shifts. However, complexes [Re(CO)_3_L1(H_2_O)]^+^ (**1**) and Re(CO)_3_L2Br (**4**) only showed weak emissions in water where relatively better emissions were obtained in acetonitrile (Additional file 1: Table S1; Figures S1, S2).

Complexes [Re(CO)_3_L1(H_2_O)]^+^ (**1**) and [Re(CO)_3_L2(H_2_O)]^+^ (**3**) were nontoxic to rat peritoneal cells up to a high concentration, such as 20.00 mg ml^−1^ where Re(CO)_3_L2Br (**4**) was toxic to same cells above 5.0 mg ml^−1^ concentrations. However every complex, at its maximum nontoxic level showed excellent staining ability for both plant and rat peritoneal cells. The binding of the compound is believed to be occurring with the large nuclei of the cells. Even though the exact binding mode or the particular substance subjected to binding cannot be distinguished, the fluorescent yield of each compound seems to be increased after binding. Better micrographs were obtained when the stained cells excited at 550 nm and the emission occurred in red region. The obtained micrographs confirm the applicability of these novel rhenium complexes as biological imaging agents.

## Additional file

**Additional file 1: Table S1.** ^1^H NMR chemical shifts (ppm) of complexes **1**–**4** in D_2_O at 25 °C. ^1^H NMR chemical shifts (ppm) of complexes **1**–**4** in D2O at 25 °C. **Figure S1.** UV VIS spectra of L1 (top), Re(CO)_3_L1Br (2, middle) and [Re(CO)_3_L1(H_2_O)]^+^ (1, bottom). **Figure S2.** UV VIS spectra of L2 (top), [Re(CO)_3_L2(H_2_O)]^+^ (3, middle) and Re(CO)_3_L2Br (4, bottom). **Figure S3.** ^1^H-^13^C HSQC spectrum of a selected region of [Re(CO)_3_L1(H_2_O)]OTf (1) (25 °C, D_2_O, shifts in ppm). **Figure S4.** ^1^H-^1^H ROESY spectrum of a selected region of [Re(CO)_3_L1(H_2_O)]OTf (1) (25 °C, D_2_O, shifts in ppm). **Figure S5.** ^1^H-^13^C HSQC spectrum of a selected region of [Re(CO)_3_L2(H_2_O)]OTf (3) (25 °C, D_2_O, shifts in ppm). **Figure S6.** ^1^H-^1^H ROESY spectrum of a selected region of [Re(CO)_3_L2(H_2_O)]OTf (3) (25 °C, D_2_O, shifts in ppm).
