# Supplementary material for: Synthesis and characterization of novel rhenium(I) complexes towards potential biological imaging applications
Source: Chem Cent J. 2016 Nov 25;10:71. doi: 10.1186/s13065-016-0218-4 (PMC5123207; doi:10.1186/s13065-016-0218-4)
Supplement: Supplementary file 1 — Additional file 1: Table S1. 1H NMR chemical shifts (ppm) of complexes 1–4 in D2O at 25 °C. 1H NMR chemical shifts (ppm) of complexes 1–4 in D2O at 25 °C. Figure S1. UV VIS spectra of L1 (top), Re(CO)3L1Br (2, middle) and [Re(CO)3L1(H2O)]+ (1, bottom). Figure S2. UV VIS spectra of L2 (top), [Re(CO)3L2(H2O)]+ (3, middle) and Re(CO)3L2Br (4, bottom). Figure S3. 1H-13C HSQC spectrum of a selected region of [Re(CO)3L1(H2O)]OTf (1) (25 °C, D2O, shifts in ppm). Figure S4. 1H-1H ROESY spectrum of a selected region of [Re(CO)3L1(H2O)]OTf (1) (25 °C, D2O, shifts in ppm). Figure S5. 1H-13C HSQC spectrum of a selected region of [Re(CO)3L2(H2O)]OTf (3) (25 °C, D2O, shifts in ppm). Figure S6. 1H-1H ROESY spectrum of a selected region of [Re(CO)3L2(H2O)]OTf (3) (25 °C, D2O, shifts in ppm). [file 13065_2016_218_MOESM1_ESM.docx]

**Supporting Information**

**Table S1.** ^1^H NMR chemical shifts (ppm) of complexes **1- 4** in D_2_O at 25 °C

| **Complex** | **H6' (d)** | **H3' (d)** | **H4' (t)** | **H5' (t)** |
| --- | --- | --- | --- | --- |
| [Re(CO)_3_L1(H_2_O)]^+^ **(1)** | 9.24 | 9.02 | 8.49 | 7.98 |
| Re(CO)_3_L1Br **(2)** | 9.25 | 9.00 | 8.48 | 7.99 |
| [Re(CO)_3_L2(H_2_O)]^+^ **(3)** | 9.29 | 9.05 | 8.53 | 8.05 |
| Re(CO)_3_L2Br **(4)** | 9.27 | 9.03 | 8.51 | 8.01 |


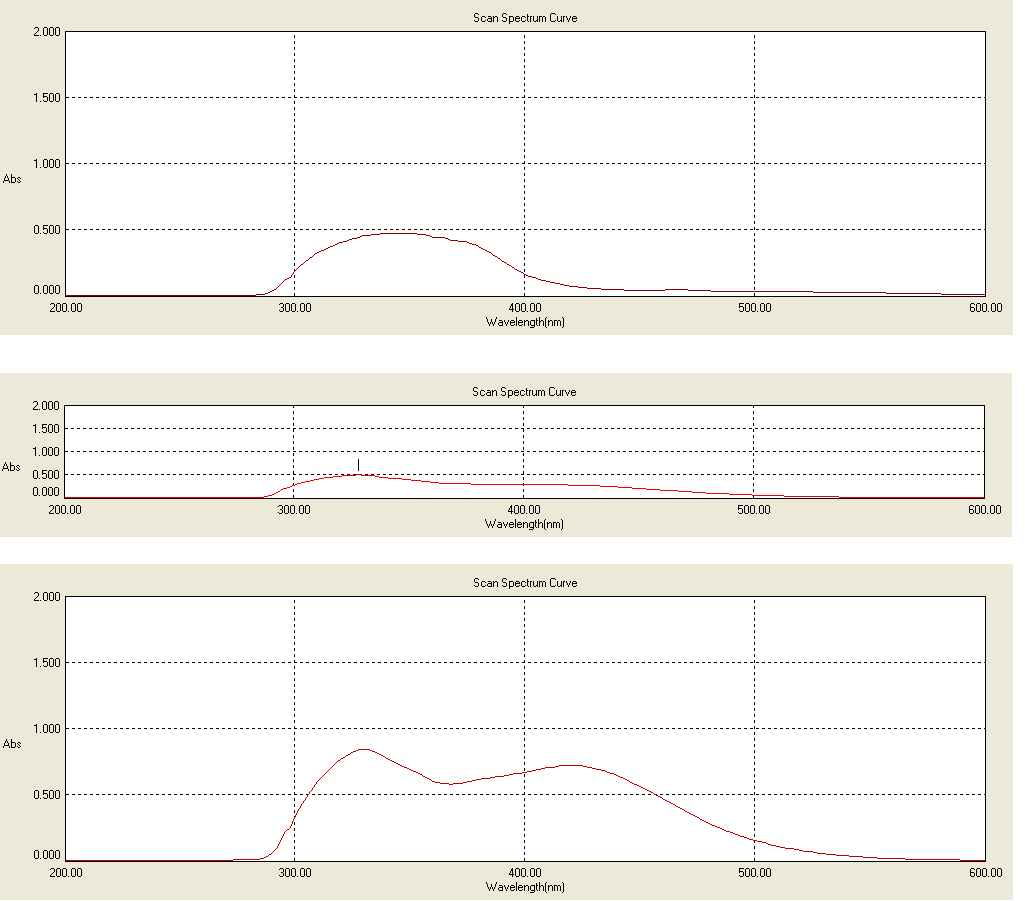


Figure S1. UV VIS spectra of L1 (top), Re(CO)_3_L1Br (2, middle) and [Re(CO)_3_L1(H_2_O)]^+^ (1, bottom).

**
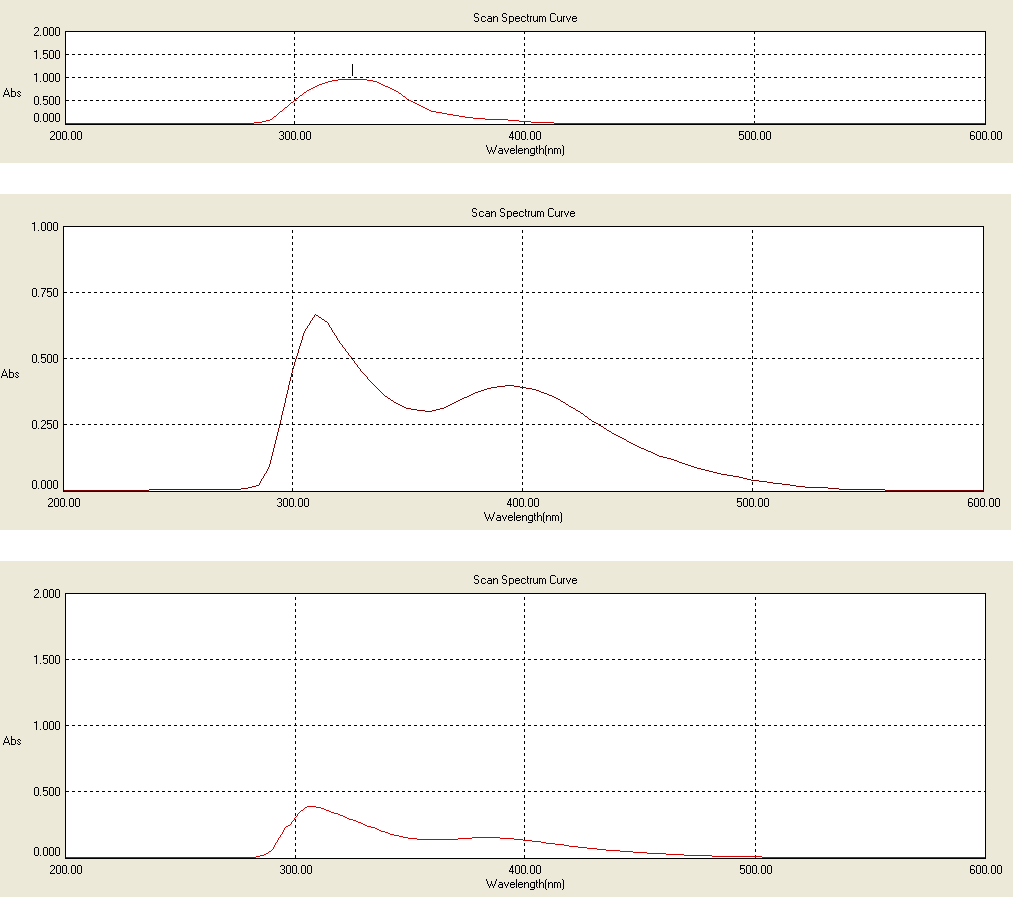
**

Figure S2. UV VIS spectra of L2 (top), [Re(CO)_3_L2(H_2_O)]^+^ (3, middle) and Re(CO)_3_L2Br (4, bottom).

**
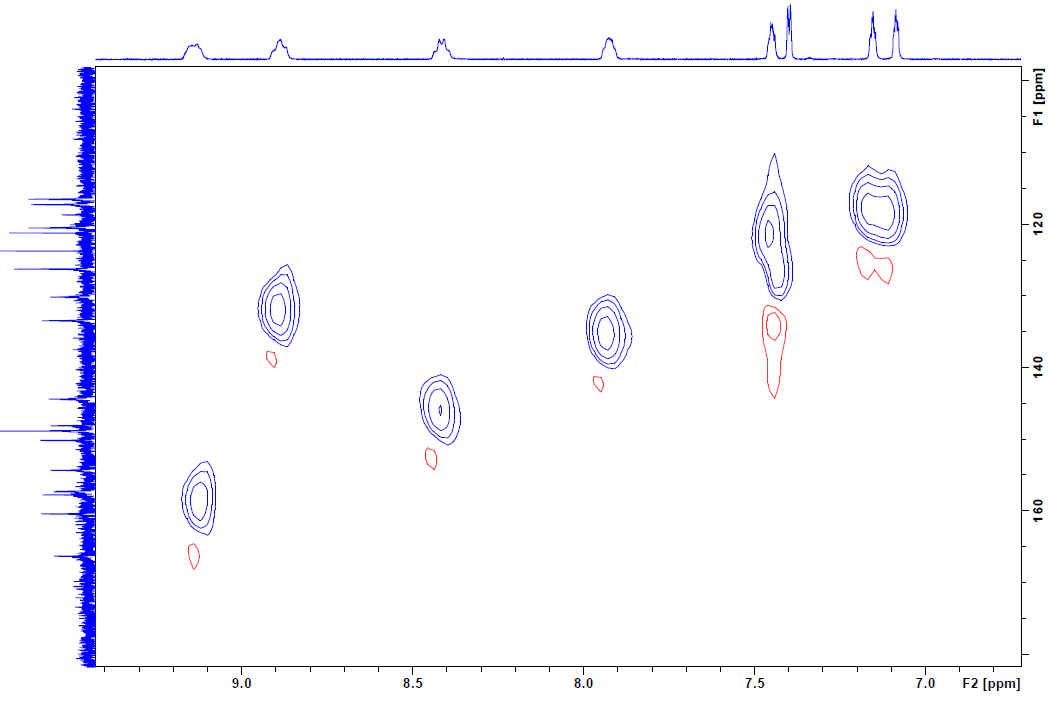
**

**Figure S3.** ^1^H-^13^C HSQC spectrum of a selected region of [Re(CO)3L1(H2O)]OTf (**1**) (25 °C, D_2_O, shifts in ppm).


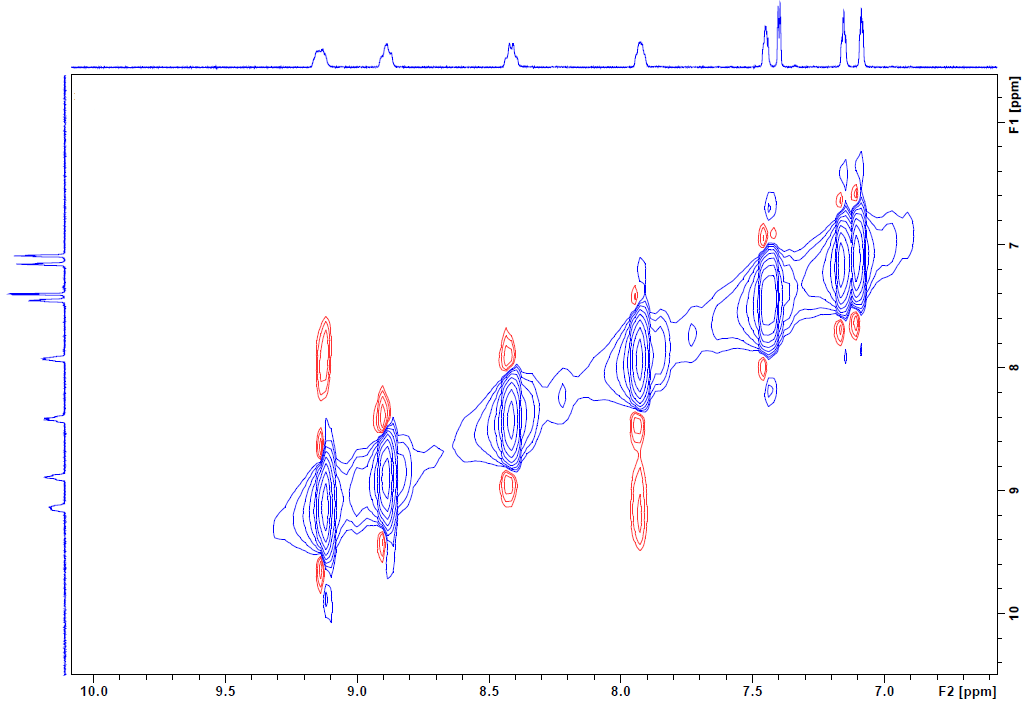


**Figure S4.** ^1^H-^1^H ROESY spectrum of a selected region of [Re(CO)3L1(H2O)]OTf (**1**) (25 °C, D_2_O, shifts in ppm).


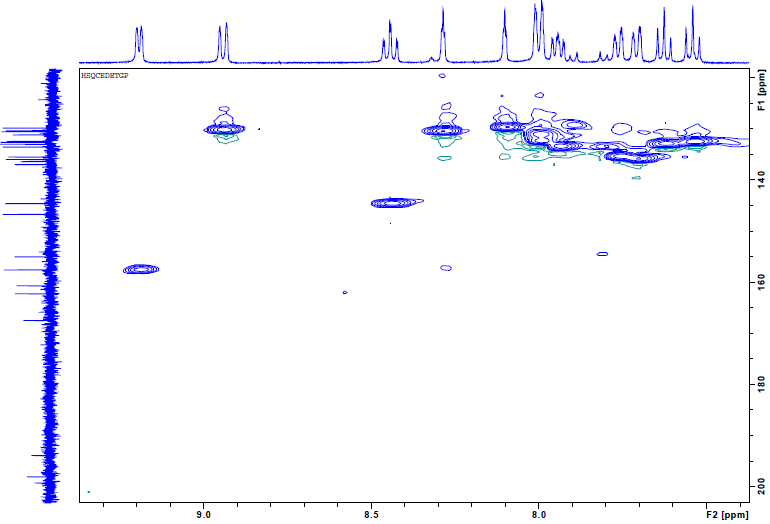


**Figure S5.** ^1^H-^13^C HSQC spectrum of a selected region of [Re(CO)3L2(H2O)]OTf (**3**) (25 °C, D_2_O, shifts in ppm).

**
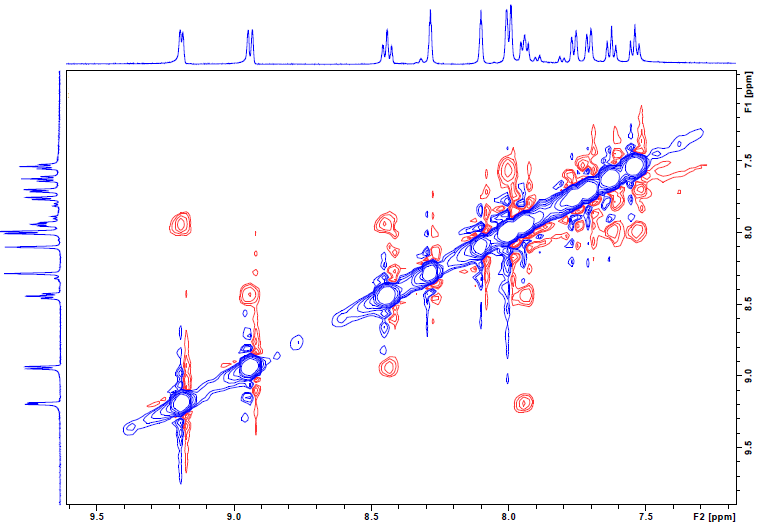
**

**Figure S6.** ^1^H-^1^H ROESY spectrum of a selected region of [Re(CO)3L2(H2O)]OTf (**3**) (25 °C, D_2_O, shifts in ppm).
